# ANTECEDENTS, OUTCOMES, AND CORRELATES OF METABOLIC HEALTH IN OLDER AGE: A MULTIDISCIPLINARY PERSPECTIVE

**DOI:** 10.1093/geroni/igac059.005

**Published:** 2022-12-20

**Authors:** Johanna Drewelies, Elisabeth Steinhagen-Thiessen, Jeremy Walston

## Abstract

Metabolic syndrome (MetS) is a cluster of risk factors for cardiovascular disease associated with reduced physical fitness, higher disease burden, and impaired cognitive functions in late life. Thus investigating antecedents, outcomes and correlates of metabolic health is a priority in order to promote healthier lifestyles and successful aging. This symposium compiles four empirical interdisciplinary studies that examine the role of metabolic health/risk on different timescales. Employing advanced modeling approaches to data obtained in large-scale studies, these reports will shed light on potential antecedents, correlates, and consequences thereof. First, Kalyani and colleagues will report data from the SPRING (Study of Physical Resilience IN Geriatrics) study on the interrelation between metabolic syndrome, glucose intolerance, and physical resilience.Second, Buchmann and colleagues examine the association of the metabolic syndrome and Lipoprotein(a) finding that hormonal aspects and in particular menopausal alterations seem to moderate theassociation between MetS and Lp(a). Third, Demuth and colleagues show how metabolic health is related to specific DNA methylation age acceleration as derived from five different epigenetic clocks, namely PhenoAge, and GrimAge.Fourth, Duezel and colleagues examine the neural correlates of metabolic risk in a sample of older adults finding that lower metabolic risk is linked to greater GMI in the prefrontal cortex. Conjointly, findings demonstrate the multifaceted nature of metabolic risk across adulthood and across different timescales. Walston will critically discuss the contributions from an aging perspective and discuss implications for future research.

